# Single institution study of the immune landscape for canine oral melanoma based on transcriptome analysis of the primary tumor

**DOI:** 10.3389/fvets.2023.1285909

**Published:** 2024-01-08

**Authors:** Isabelle F. Vanhaezebrouck, Kimaya M. Bakhle, Carlos R. Mendez-Valenzuela, L. Tiffany Lyle, Kristoph Konradt, Matthew L. Scarpelli

**Affiliations:** ^1^Radiation Oncology, Small Animal Medicine, College of Veterinary Medicine Purdue University, West Lafayette, IN, United States; ^2^College of Veterinary Medicine, Cornell University, New York, NY, United States; ^3^Pathology Cook Research Inc., West Lafayette, IN, United States; ^4^Comparative Pathology, College of Veterinary Medicine, Purdue University, West Lafayette, IN, United States; ^5^School of Health Sciences, Purdue University, West Lafayette, IN, United States

**Keywords:** dog, oral melanoma, immune landscape, transcriptome, cancer

## Abstract

**Introduction:**

Understanding a tumor’s immune context is paramount in the fight against cancer. Oral melanoma in dogs serves as an excellent translational model for human immunotherapy. However, additional study is necessary to comprehend the immune landscape of dog oral melanomas, including their similarity to human melanomas in this context.

**Methods:**

This retrospective study utilizes formalin-fixed paraffin-embedded (FFPE) tissue samples to analyze RNA sequences associated with oral melanoma in dogs. Nanostring Technologies was used for conducting RNA sequencing. The focus is on understanding the differences between melanoma tumors restricted to the oral cavity (OL) and the same primary oral tumors with a history of metastasis to the lymph nodes or other organs (OM). Normal buccal mucosa samples are also included as a normal tissue reference.

**Results:**

In the OM patient group, gene signatures exhibit significant changes relative to the OL patient group, including significantly decreased expression of S100, BRAF, CEACAM1, BCL2, ANXA1, and tumor suppressor genes (TP63). Relative to the OL tumors, the OM tumors had significantly increased expression of hypoxia-related genes (VEGFA expression), cell mobility genes (MCAM), and PTGS2 (COX2). The analysis of the immune landscape in the OM group indicates a shift from a possible “hot” tumor suppressed by immune checkpoints (PDL1) to significantly heightened expression not only of those checkpoints but also the inclusion of other immune blockades such as PD1 and IDO2. In addition, the OM group had significantly reduced expression of Toll-like receptors (TLR4) and IL-18 relative to the OL group, contributing to the tumor’s immune escape. Additionally, signs of immune cell exhaustion are evident in both the OM and OL groups through significantly increased expression of TIGIT relative to normal tissue. Both the OM and OL groups had significantly increased expression of the immune cell marker CD4 expression relative to normal tissue. Further, CD4 expression significantly decreased in OM relative to OL; however, this study cannot determine the specific cell types expressing CD4 in OM and OL tumors.

**Discussion:**

This preliminary study reports significant changes in gene expression for oral melanoma between canine patients with localized disease relative to those with metastatic disease. In the future, a more in-depth investigation involving immunohistochemistry analysis and single-cell RNA expression is necessary to confirm our findings.

## Introduction

Oral melanoma is relatively common in canine companions, accounting for 7% of all malignancies. It is known for its aggressive nature (1). The cancer tends to metastasize first to the regional lymph nodes, then to the lungs, and less commonly to the liver, brain, and adrenal glands. The prognosis for affected dogs depends on the stage (TNM classification), pathology grade, mitotic index of cells, and Ki-67 expression (2). Pathologists have drawn comparisons between oral melanoma in canine and human melanoma due to similar gene dysregulations in the NRAS, AKT, and PTEN pathways, mutations in c-Kit (found in about 10% of cases), and overexpression of Cox-2. However, it’s worth noting that Braf mutations are highly prevalent in human melanoma and occur less frequently in canine melanoma (3–7).

The oral cavity is the most common location for melanoma in dogs, and it can affect the lips, oral mucosa, the tongue, or the jawbone (maxilla or mandible). Treatment usually involves a multidisciplinary approach, which includes surgical excision to ensure healthy margins and radiotherapy. Chemotherapy has shown disappointing results similar to those seen in human subjects (8). Overall survival for most dogs with advanced local disease (tumor size >2 cm) or disseminated form is poor, with an average of 7–10 months (9, 10)_._

For the last 15 years, the veterinary community has been focused on researching and making progress in immunotherapy (11–14). Cancer cells can suppress the body’s immune response and utilize surrounding cells, including immune cells, to promote their own growth and survival gain. Promoting an adequate immune response is paramount in the fight against cancer (15). Veterinary oncologists cannot achieve therapeutic improvement for these patients without understanding the complex interaction between cancer cells and surrounding immune cells.

In a preliminary veterinary pathology study using immunohistochemistry (IHC), the lymphocyte density in 32 oral tumors was analyzed, and it was found that a low B cell count was associated with a better prognosis. However, the authors could not comment on the T cell population based on its diversity, including pro-immune versus immunosuppressive Tregs (16). In addition, a recent study in canine melanoma reported on 25 biopsies using IHC and RNA extraction along with qualitative real-time polymerase chain reaction techniques to analyze the expression of immunosuppression markers FoxP3, IDO, and CTLA4. An analysis of all samples showed gene and protein expression correlated with poor prognoses (17).

Galon et al. (18) introduced the concept of Immunoscore^®^ while studying human colorectal cancers in 2006. He quantified different populations of lymphocytes, CD8, CD3, and CD45R, their collocation at the center of the tumor versus the periphery, and their functional immune orientation (based on gene expression profiling). A scoring scheme was also introduced, establishing a patient’s prognosis based on the immune landscape. The classification was more accurate than standard staging and pathological classification for predicting patient survival. The concept has gained international recognition and has become a benchmark for predicting prognosis.

Preliminary work on human skin melanoma reveals a complex immune landscape. For human melanoma patients treated with immune checkpoint inhibitor ipilimumab, a prominent CD8 infiltration and PDL1 negative status for lymph node metastasis is considered a favorable prognostic factor (19)_._

Human pathologists have increasingly employed digitalization and artificial intelligence software to facilitate the estimation of large-scale cell counts. A comprehensive understanding of cell type, density, count, and immune cells distribution within the tumor is crucial (20). Moreover, coupling this information with genomic analyses encompassing chemokines, checkpoints, and interferon activity can provide a more insightful evaluation of the tumor immune landscape and microenvironment. This integrated approach holds significant promise in accurately assessing a patient’s prognosis and guiding treatment strategies. Ultimately, it could pave the way for a personalized treatment tailored to the patient’s cancer’s unique characteristics (21). Similar analyses in veterinary pathology could benefit our canine patients to determine the progression of the disease.

This single-institution clinical research study aimed to characterize the immune landscape of oral canine melanoma. We present an analysis of the immune contexture within the tumor microenvironment based on Nanostring technology (800 genes- IO canine- Ncounter^®^, Nanostring, Seattle, WA, 98109, United States) derived from 18 FFPE oral tumors. Dogs are characterized by spontaneous disease, a natural progression of cancer growth, and adaptation to the host environment. Understanding the immune context for canine melanoma could stimulate further research supporting translation to human melanoma (22).

## Materials and methods

### Tissue samples

Eighteen primary melanoma tumor samples and five normal tissue samples from buccal mucosa were procured from the pathology laboratory at Purdue University Veterinary College. All melanoma samples came from the primary oral tumor. All normal tissue samples were derived from the oral labial mucosa in healthy dogs. FFPE samples were obtained from biopsy, surgical excision, or autopsy between 2000 and 2020. Previous studies at our institution and others have reported the pathology approach for analyzing melanoma with IHC identification and classification (23, 24). To ensure accuracy, a board-certified pathologist (TL) performed a second review for each sample included in the study. Tumors were classified as local malignant (OL) if no evidence of metastasis was mentioned (staging obtained from the referring veterinarian or data from the pathology laboratory) or oral malignant (OM) for primary tumors with known metastases. Given the specific nature and objectives of this study, clinicopathological characteristics were not included in the analysis.

### Sample processing and RNA sequencing technique

For each sample, between 50 and 100 ng was shipped to Nanostring^®^ for RNA extraction. The extracted RNA was solubilized and mixed in a hybridization solution. The 50 kb segment of interest was hybridized to a capture probe, and the distal part (50 kb) was linked to a fluorescent probe. Each type of fluorescence obtained was associated with a unique gene. The Nanostring^®^ canine Immuno-Oncology (IO) chip targeting 800 genes related to cancer and immune markers was used for this analysis.

Following 24 h of hybridization, the samples were purified and placed in a cartridge. The fluorescent probes were immobilized using a current, and the samples were then ready for optical scan counting. The resulting data were presented approximately as the amount of fluorescence relative to housekeeping genes.

### Normalization and statistical analysis

Data were normalized using the geNorm algorithm to identify suitable housekeeping genes. After removing potential candidates such as G6PD, OAZ1, TBC1D10B, TFRC, and UBB, the final set of housekeeping genes included ABCF1, DNAJC14, ERCC3, GUSB, MRPL19, NRDE2, POLR2A, PSMC4, PUM1, SDHA, SF3A1, STK11IP, TBP, TLK2, and TMUB2.

For Statistical analysis, a non-paired *t*-test with a false discovery rate *post-hoc* test was performed to determine significant differences among samples. Quality control metrics employed by the NanoString platform encompass Imaging QC, Binding Density QC, Positive Control Linearity, and Limit of Detection QC. These internal controls assess the technical success of the assay.

The quantification of targets was achieved through direct digital counting of a hybridized fluorescent barcode, which was bound to a streptavidin-coated imaging surface. These raw counts were normalized to housekeeping targets, and data were expressed as Log2 normalized counts.

### Data analysis

**Figure 1:**
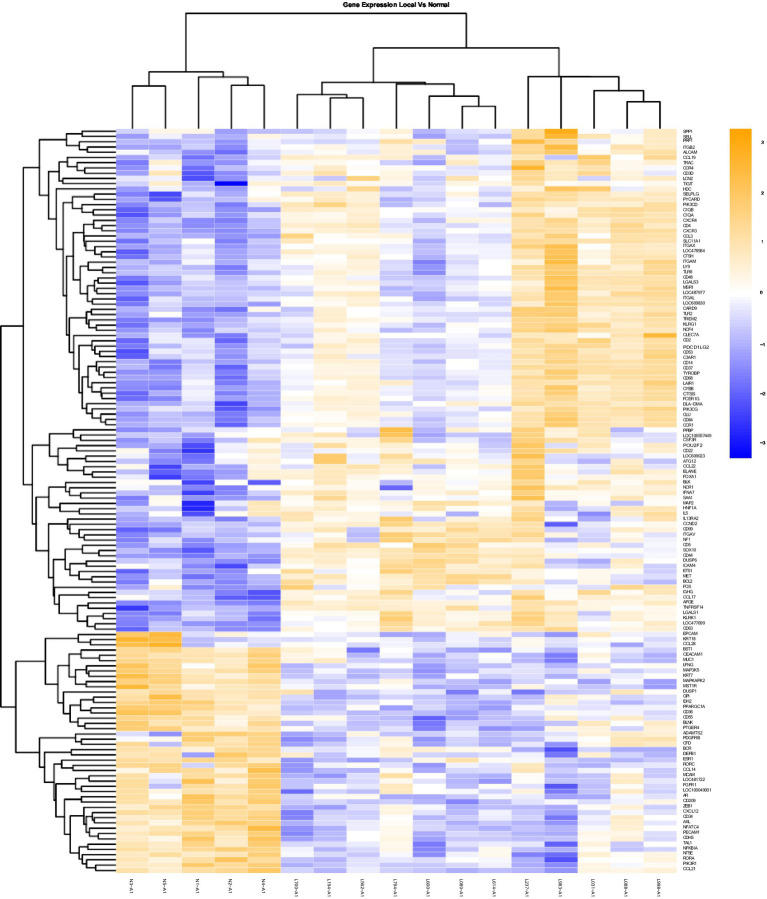
Gene Expression Comparison Patterns of Local (OL) Vs. Normal Oral Mucosa: 142 genes were found significantly expressed in the comparison of Local vs Normal mucosa. The list of genes is presented on the right “Y” axis of the heat map. Patients are indicated on the horizontal plane. Samples that start with an “N” come from normal dogs, and those that start with an “L” are from patients with local tumors. The color blue indicated low expression compared to high expression by orange.

**Figure 2:**
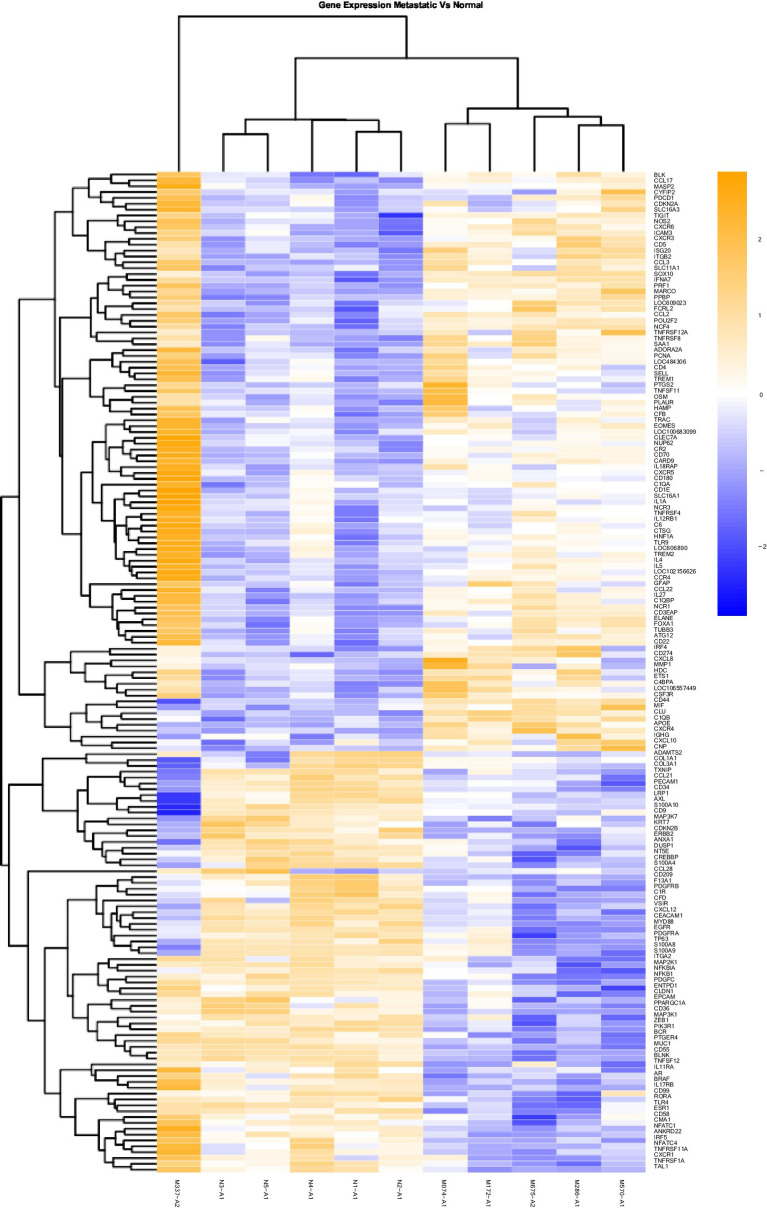
Gene Expression Comparison Patterns of Metastatic (OM) Vs. Normal Oral Mucosa: 171 genes were found significantly expressed in the comparison of Metastatic vs Normal mucosa. The list of genes is presented on the right “Y” axis of the heat map. Patients are indicated on the horizontal plane. Samples that start with an “N” come from normal dogs, and those that start with an “M” are from patients with metastatic tumors. The color blue indicated low expression compared to high expression by orange.

**Figure 3:**
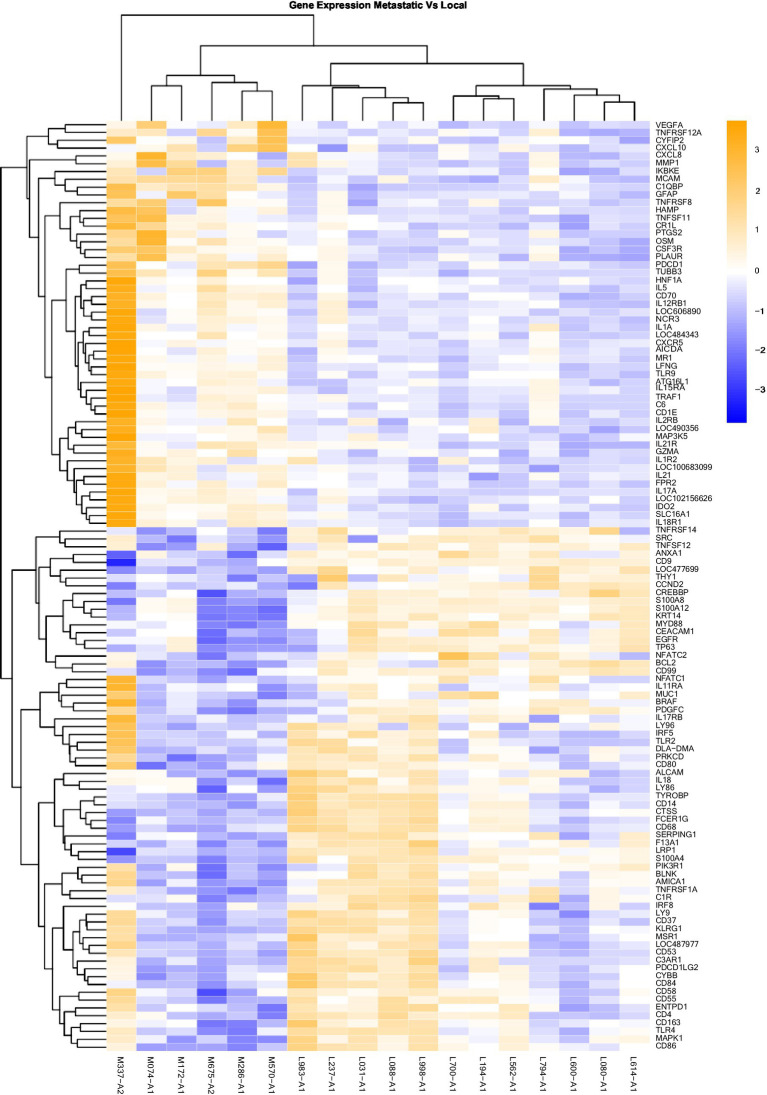
Gene Expression Comparison Patterns of Metastatic (OM) Vs. Local (OL) Tumors: 119 genes were found significantly expressed in the comparison of Metastatic vs Normal mucosa. The list of genes is presented on the right “Y” axis of the heat map. Patients are indicated on the horizontal plane. Samples that start with an “M” come from metastatic dogs, and those that start with an “L” are from patients with local tumors. The color blue indicated low expression compared to high expression by orange.

A set of genes representing a potentially clinically relevant immune profile, cellular and microenvironment dysregulation, was identified through gene expression analysis. The significance of the expression was determined by assessing fold changes and adjusted *p*-values. The visualization of the data was accomplished by generating heat maps using R software [R Foundation for Statistical Computing, Vienna, Austria, Version 4.2.3.] with the utilization of the “heatmap” function as illustrated in Figures 1–3.

Furthermore, the significance of the expression levels was depicted through tables comparing overexpressed versus under-expressed genes. This comparison was made across various scenarios: local tumor (OL) versus normal buccal mucosa tissues (Table 1), local tumor with known metastasis (OM) versus normal buccal mucosa tissues (Table 2), and local tumor with known metastasis (OM) versus local tumor known to remain at the local level (OL) (Table 3). For each tissue sampled, the biopsy included the epithelial part and the stromal part. Statistically significant differences across groups was determined based on a ±1.5-fold threshold indicating differential changes with an adjusted *p*-value 0.05.

**Table 1:** Significantly expressed genes *of* local (OL) vs. normal.

Significantly expressed genes local (OL) vs. normal				
Cell type	Gene symbol	Protein description	Fold	*P*-value adj.
T cell	CD4	CD4 molecule	15.4079	0.0000646
NK cell	KLRG1	Killer cell lectin-like receptor subfamily G, member 1	11.231	0.000627
B cell	BLK	B lymphoid tyrosine kinase	3.35318	0.034682
	TYROBP	TYRO protein tyrosine kinase binding protein	5.18089	0.001619
Macrophages	CD14	CD14 molecule	5.8061	0.002133
	CD68	CD68 molecule	4.89344	0.009834
	CD84	CD84 molecule	13.3984	0.000931
	CD99	CD99 molecule, Protein MIC2	1.82053	0.032208
Cytokines	IFNA7	Interferon, alpha 7	4.59887	0.000105
Melanoma cells	BCL2	B-cell CLL/lymphoma 2	2.46065	0.002709
	CEACAM1	Carcinoembryonic antigen-related cell adhesion molecule 1	−3.42594	0.006294
	MCAM	Melanoma cell adhesion molecule	−2.39599	0.023284
	SOX10	SRY (sex determining region Y)-box 10	26.6667	0.000106
Immune checkpoints	TIGIT	T cell immunoreceptor with Ig and ITIM domains	3.05555	0.04822
	PDCD1LG2	Programmed cell death ligand 2	5.49496	0.006176

The genes of local vs. normal that show an adjusted *p*-value below 0.05 with a fold change considered relevant (±1.5) are shown in the table. In addition, the possible associated cell types expressing the gene, gene symbol, and encoded protein description are presented.

**Table 2:** Significantly expressed genes *of* metastatic (OM) vs. normal.

Significantly expressed genes metastatic (OM) vs. normal				
Cell type	Gene symbol	Protein description	Fold	*P*-value adj.
T cell	CD4	CD4 molecule	4.38595	0.015951
	OSM	Oncostatin M	40.1851	0.000374
NK cell	NCR3	Natural cytotoxicity triggering receptor 3	1.96637	0.049649
B cells	BLK	B lymphoid tyrosine kinase	4.32539	0.019839
Macrophages	CD99	CD99 molecule, MIC2	−2.3836	0.008101
	MIF	Macrophage migration inhibitory factor	2.59845	0.023702
	TLR4	Toll-like receptor 4	−2.79411	0.021418
	TLR9	Toll-like receptor 9	2.61574	0.012437
Cytokines	IFNA7	Interferon, alpha 7	7.10178	0.0000242
	IRF4	Interferon regulatory factor 4	6.83485	0.00872
	IRF5	Interferon regulatory factor 5	−4.06668	0.008101
	ISG20	Interferon stimulated exonuclease gene 20kDa	6.39423	0.012437
Melanoma cells	ANXA1	Annexin A1	−5.8559	0.002254
	BRAF	v-raf murine sarcoma viral oncogene homolog B1	−2.21539	0.0000242
	CEACAM1	Carcinoembryonic antigen-related cell adhesion molecule 1	−9.41297	0.000127
	ICAM3	Intercellular adhesion molecule 3	3.7162	0.035191
	NOS2	Nitric oxide synthase 2, inducible	3.22223	0.038384
	PCNA	Proliferating cell nuclear antigen	1.91176	0.049501
	PTGS2	Prostaglandin-endoperoxide synthase 2 (prostaglandin G/H synthase and cyclooxygenase-2)	7.92719	0.003146
	S100A10	S100 calcium binding protein A10	−5.74469	0.032769
	S100A4	S100 calcium binding protein A4	−5.11119	0.000385
	S100A8	S100 calcium binding protein A8	−11.5099	0.012437
	S100A9	S100 calcium binding protein A9	−14.2991	0.007404
	SOX10	SRY (sex determining region Y)-box 10	48.6906	0.0000425
	TP63	Tumor protein p63	−4.62351	0.018215
Immune checkpoints	PDCD1	Programmed cell death 1	3.24433	0.010195
	CD274	Programmed cell death ligand 1 (PDL1)	3.57006	0.025641
	TIGIT	T cell immunoreceptor with Ig and ITIM domains	5.60184	0.006844

The genes of metastatic vs. normal that show an adjusted *p*-value below 0.05 with a fold change considered relevant (±1.5) are shown in the table. In addition, the possible associated cell types expressing the gene, gene symbol, and encoded protein description are presented.

**Table 3:** Significantly expressed genes *of* metastatic (OM) vs. local (OL).

Significantly expressed genes metastatic (OM) vs. Local (OL)				
Cell type	Gene symbol	Protein description	Fold	*P*-value adj.
T cell	CD4	CD4 molecule	−3.51299	0.015868
	IL18	Interleukin 18 (interferon-gamma-inducing factor)	−2.37245	0.048949
	OSM	Oncostatin M	9.48633	0.003424
	THY1	Thy-1 cell surface antigen	−2.20355	0.036482
NK cell	GZMA	Granzyme A (granzyme 1, cytotoxic T-lymphocyte-associated serine esterase 3)	2.53334	0.036301
	KLRG1	Killer cell lectin-like receptor subfamily G, member 1	−9.5645	0.001186
	NCR3	Natural cytotoxicity triggering receptor 3	1.93525	0.027144
B cells	TYROBP	TYRO protein tyrosine kinase binding protein	−4.22687	0.003424
Macrophages	CD14	CD14 molecule	−4.86962	0.003424
	CD163	CD163 molecule	−2.55398	0.017287
	CD68	CD68 molecule	−4.35219	0.011847
	CD84	CD84 molecule	−6.68181	0.005718
	CD86	CD86 molecule	−4.75581	0.001295
	TLR4	Toll-like receptor 4	−2.7451	0.011192
	TLR9	Toll-like receptor 9	2.29442	0.009216
Major histocompatibility complex (class 1)	MR1	Major histocompatibility complex, class I-related	1.7485	0.047368
Cytokines	IRF5	Interferon regulatory factor 5	−3.23611	0.011934
	IRF8	Interferon regulatory factor 8	−2.55556	0.035517
Melanoma cells	ANXA1	Annexin A1	−2.83874	0.022851
	BCL2	B-cell CLL/lymphoma 2	−3.46134	0.00013
	BRAF	v-raf murine sarcoma viral oncogene homolog B1	−1.68219	0.000982
	CEACAM1	Carcinoembryonic antigen-related cell adhesion molecule 1	−2.74756	0.016576
	MCAM	Melanoma cell adhesion molecule	3.01003	0.004324
	PTGS2	Prostaglandin-endoperoxide synthase 2 (prostaglandin G/H synthase and cyclooxygenase-2)	5.63185	0.003637
	S100A12	S100 calcium binding protein A12	−5.39106	0.0396
	S100A8	S100 calcium binding protein A8	−5.05685	0.046783
	S100A9	S100 calcium binding protein A9	−4.83414	0.050571
	TP63	Tumor protein p63	−3.77941	0.018472
	VEGFA	Vascular endothelial growth factor A	5.24266	0.006493
Immune checkpoints	PDCD1 (PD1)	Programmed cell death 1	3.97031	0.001295
	IDO2	Indoleamine 2,3-dioxygenase 2	2.83516	0.004725
	PDCD1LG2	Programmed cell death ligand 2	−5.95454	0.003424

The genes of metastatic vs. local that show an adjusted *p*-value below 0.05 with a fold change considered relevant (±1.5) are shown in the table. In addition, the possible associated cell types expressing the gene, gene symbol, and encoded protein description are presented.

## Results

### Population characteristics

A total of 18 canine patients with a documented history of malignant melanoma between 2000 and 2020 were included in this study as described in Table 4. The average age of these patients was 10.5 years. Of the 18 patients, nine were castrated males, one was an intact male, six were spayed females, and two were intact females. Regarding breed distribution, most patients were of mixed breed (8/18), followed by three Golden Retrievers, two Labrador retrievers, two Schnauzers, one Cocker Spaniel, one Poodle, and one Scottish terrier (Table 4).

**Table 4:** Clinical data from tissue samples.

Classification table of patient pathology							
ID	Species	Breed	Sex	Age	Tissue location	Mitotic index	Metastasis
L031-A1	Canine	Poodle	M	10	Left canine gingiva	0	No
L080-A1	Canine	Golden Retriever	MN	7	Hard palate	3	No
L088-A1	Canine	Schnauzer	FS	11	Oral Mucosa	1	No
L194-A1	Canine	Mixed Breed	FS	13	Right maxillary labial gingiva	0	No
L237-A1	Canine	Mixed Breed	MN	10	Hard palate	2	No
L562-A1	Canine	Mixed Breed	F	6	Oral Mucosa	0	No
L600-A1	Canine	Labrador Retriever	MN	9	Right Lip and hard palate	2	No
L614-A1	Canine	Mixed Breed	FS	6	Hard palate	1	No
L700-A1	Canine	Golden Retriever	MN	11	Oral Mucosa	0	No
L794-A1	Canine	Mixed Breed	MN	12	Lip	3	No
L983-A1	Canine	Mixed Breed	MN	11	Base of tongue	1	No
L998-A1	Canine	Mixed Breed	MN	9	Oral Mucosa	0	No
M074-A1	Canine	Labrador Retriever	MN	11	Left Mandibula	12	Lungs, Tracheobronchial lymph node, Pulmonary artery
M172-A1	Canine	Coker Spaniel	MN	12	Hard palate	15	Lymph node, Lung, Liver, Kidney, Heart, Esophagus
M286-A1	Canine	Golden Retriever	FS	12	Tonsils	15	Regional lymph nodes, lung
M337-A2	Canine	Mixed Breed	FS	13	Left Maxilla, bone involvement	40	Lungs
M570-A1	Canine	Schnauzer	F	14	Jaw region and pharynx	15	Lungs
M675-A2	Canine	Scottish Terrier	FS	12	Right Maxilla, Peri-orbital	12	Lung, Sub Mandibular and medial ileac lymph nodes, mesocolon
N1-A1	Canine	NA	NA	NA	Oral labial mucosa	NA	No
N2-A1	Canine	NA	NA	NA	Oral labial mucosa	NA	No
N3-A1	Canine	NA	NA	NA	Oral labial mucosa	NA	No
N4-A1	Canine	NA	NA	NA	Oral labial mucosa	NA	No
N5-A1	Canine	NA	NA	NA	Oral labial mucosa	NA	No

Clinical information from the 18 pathology samples, ID samples that start with an L correspond to local tumors, ID samples that start with an M correspond to metastatic samples, and ID samples that start with N correspond to normal tissue samples. Breed, sex, age, and metastasis status are presented with the primary tumor location for all samples. The mitotic index corresponds to the number of cells undergoing mitosis per ten histopathology high power fields as indicated per pathology report from each patient sample. NA, Not available.

The oral primary tumor location is reported in Table 4. It was observed that the mitotic index was higher than 10 (per 10 Hpf) for oral tissues with a known history of metastasis. In contrast, for local tumors without available information on metastasis, the mitotic index was no more than three. Overall, 12 OL cavity samples were compared to six OM samples and five standard canine buccal tissue samples as shown in Table 4.

Heat maps providing a comparison of marker expressions that were significantly different between the three different groups are shown in Figures 1–3. In addition, Tables 1–3 summarize clinically relevant markers with significant changes in expression. Supplementary Tables S1–S3 show changes in expression for clinically relevant markers regardless of significance level.

### Melanoma cell markers

Melan A, tyrosinase, and S100 are commonly used genes by pathologists to identify melanoma-associated proteins. The genes encoding proteins S100 A4, S100A8, S100A9, S100 A10, and S100A12 showed significantly decreased expression in OM relative to normal tissue (Table 1). Conversely, the expression of Sox 10 was significantly elevated in both OL and OM groups relative to normal tissue. Sox 10 is present during embryonal life and is a nuclear transcriptional factor in melanogenesis (25).

BRAF is significantly under-expressed in OL tumors compared to normal tissues (Table 1) and in OM tumors compared to normal samples (Table 2). When comparing OL tumors vs. OM tumors, BRAF was significantly under-expressed in OM compared with OL (Table 3). BRAF inhibitors are frequently used for human skin melanoma. However, regarding the under-expression of BRAF in canine oral melanoma, BRAF-targeted therapy may not be an option.

CEACAM1 (carcinoembryonic antigen-related cell adhesion molecule 1) is significantly under-expressed in OL vs. normal tissues (Table 1). Furthermore, the decreased expression becomes even more significant when comparing OM vs. normal tissue (Table 2). These findings suggest a progressive decrease in this marker when transitioning from OL to OM phenotype. This tumor-associated antigen serves as a prognostic biomarker in several human cancers; for example, low CEACAM expression is linked with poor survival for patients with clear cell renal carcinoma (26).

DMBT1 was under-expressed in all melanomas in this study, albeit the decreases were not statistically significant (Supplementary Tables S1–S3). In human patients, DMBT1 is present in anorectal melanoma but not in the skin and functions as a suppressor gene. DMBT1 gene deletion or loss of function is associated with tumor progression in glioblastoma, medulloblastoma, and lung, gastric, and colorectal tumors (27).

NOS2 was elevated in OM samples compared to normal tissue (Table 2). Elevated NOS2 in human melanoma amplifies P13/AKT, HIF, and Ras pathways, TGF beta expression, and lower immune function. Moreover, NOS2 gene expression has been linked to a poor prognosis (28).

For OM, Annexin (ANXA1) apoptosis marker expression was significantly lower relative to normal tissue (Table 2) and OL samples (Table 3). The BCL2 apoptosis regulator was significantly overexpressed in OL relative to normal tissue but significantly under expressed in OM relative to OL. Significantly less expression of the tumor suppressor TP63 was evident in OM relative to normal tissue (Table 2) and OL (Table 3). A comparison between OM and OL (Table 3) showed a significant elevation in expression for PTGS2 (Cox2) in OM. In addition, PTGS2 was also found significantly elevated in OM vs. normal tissue (Table 2) but not in OL vs. normal tissue (Supplementary Table S1). The angiogenesis marker, VEGFA, expression was downregulated in OL vs. normal tissue (albeit not significant change; Supplementary Table S1) and VEGFA was upregulated in OM vs. normal tissue (albeit not significant change; Supplementary Table S2). VEGFA was significantly upregulated when comparing OM vs. OL (Table 3). The cell mobility marker, MCAM, was significantly downregulated in OL vs. normal tissue (Table 1) and upregulated in OM vs. normal tissue (albeit not significant change; Supplementary Table S2). MCAM was significantly upregulated when comparing OM vs. OL (Table 3). To summarize, the OM expression pattern relative to the OL expression pattern, included reduced apoptosis, tumor suppressor gene repression, promotion of angiogenesis, and increased cell mobility, which are typically associated with disease progression and are considered hallmarks of cancer (15).

### Immune landscape

#### Immune cell markers

##### T cell markers

The analysis revealed a significantly increased expression of CD4 in both OM and OL groups compared to normal tissue. However, CD4 and TH1 are significantly under-expressed in OM compared to OL (Table 3). The source of upregulated CD4 in OM and OL relative to normal tissues cannot be determined from this analysis but could be due to increased T effectors, T regulatory cells, and or neutrophils. More definitive analysis would require single-cell RNA analysis. The reasons for upregulated CD4 may also be different between OL and OM groups.

The expression of OSM (Oncostatin M) was significantly elevated in OM relative to OL (Table 3). Previous studies have reported a synergistic association between IL1Beta, IL-6, and OSM in human breast cancer, which is linked to a poor prognosis (29). Regarding the expression levels of pro-immune factors, analysis revealed non-significant expression of IL-2, IL-15, interferon beta1(IFNB1), and interferon gamma. Only IL-18 had a significant expression in OM compared to OL (Table 3).

##### NK cell makers

KLRG1 was significantly overexpressed in OL vs. normal samples (Table 1). However, KLRG1 was not significantly overexpressed in OM vs. normal samples and KLRG1 was significantly under-expressed in OM compared to OL (Table 3). KLRG1 acts as an inhibitory signal (checkpoint) for NK and T cells but is also expressed in Tregs (30). Granzyme A activity was significantly increased for OM relative to OL (Table 3). Additionally, expression of NCR3 was significantly higher in OM compared to normal and OL samples (Tables 2, 3). NCR3 is of prime importance for maintaining the cytotoxic function of NK cells (31). The increased Granzyme A and NCR3 in OM suggests a cytotoxic phenotype of NK cells in OM, whereas NK activity appears to be inhibited in OL, based on the increased expression of KLRG1.

##### B cell markers

The BLK gene is responsible for B cell proliferation and is significantly overexpressed in OM and OL relative to normal tissue (Tables 1, 2). Notably, the tertiary lymphoid structure has been associated with a positive response to immunotherapy checkpoint inhibitors in numerous cancer types (32).

##### Macrophage makers

Relative to normal tissues, OL tumors had significant upregulation of macrophage markers (CD14, CD68, CD84, and CD99). On the contrary, for OM tumors, these macrophage markers were not significantly upregulated relative to normal tissues (Table 2 and Supplementary Table S2). Further analysis reveals a notable significant decrease in the expression of markers associated with macrophages when comparing OM vs. OL tumors (Table 3). This includes decreased expression of both M1 (e.g., CD14) and M2 (e.g., CD163) markers in OM relative to OL. These results suggest OM tumors may contain fewer macrophages than OL tumors.

IRF4 expression has been associated with the presence and activity of M2 macrophages in the tumor, which are known to promote tumor development and immunosuppression (33). In OM, there was significantly increased IRF4 expression relative to normal tissue (Table 2) but not in OL vs. normal tissue. IRF4 is known to act as a negative regulator of TLR synthesis (34). In OM, we also observed significantly reduced expression of TLR 4 relative to normal tissue and OL (Tables 2, 3). In addition, reduced expression of IRF5, associated with interferon synthesis, was observed in OM relative to OL and normal tissue (Tables 2, 3). Macrophage inhibitory factor (MIF) levels significantly increased in OM vs. normal tissue (Table 2) but not in OL vs. normal tissue (Table 1). MIF is associated with progressive disease in human melanomas and is a potential target for advanced stages. Additionally, in OM, TLR9 expression is significantly increased relative to OL and normal tissue (Tables 2, 3). TLR9 has been linked to myeloid-derived suppressor cell (MDSC) activity in several cancers (35). Overall, the transcriptome analysis for macrophages suggests for advanced oral melanomas, there is less macrophage infiltration within the tumor and a transition from a pro-inflammatory, immune status toward an immunosuppressive status.

#### Immune checkpoints

Immune Checkpoints are expressed by different cell types such as T cells (PD1, TIGIT), macrophages, dendritic cells, and tumor cells (PDL1 and IDO2). The current methodology associated with this study cannot differentiate which cells are leading to changes in expression. Transcriptome analysis of immune checkpoints revealed a significant increase in the expression of PDL1 (gene CD274 in Table 2) in OM tumors compared to normal tissues. PDL1 is a transmembrane protein expressed by tumors and immune cells (lymphocytes Treg, macrophages type 2, MDSCs). It acts as a negative regulator of tumor rejection by the adaptive T-cell response. PD1 expressed on T cells, is the receptor for PDL1 and is also significantly overexpressed in OM vs. normal (Table 2) and OM vs. OL (Table 3). PDCD1LG2 (PDL2) is significantly over-expressed in OL compared to normal tissues (Table 1) but not significantly over-expressed in OM compared with normal tissues. Furthermore, PDL2 is significantly under-expressed in OM vs. OL (Table 3), which could be associated with immune exhaustion in OM tumors. PDL2 expression has been associated with immunosuppression and it is generally expressed on antigen presenting cells as well as tumor cells (36). CTLA4 was overexpressed in OM relative to both OL and normal tissue, but the differences were not statistically significant (Supplementary Tables S1–S3).

IDO1 and IDO2 are members of tryptophan catabolic pathways expressed by tumor cells and tumor microenvironment cells (dendritic cells, macrophages, endothelial cells, tumor-associated Fibroblasts). These proteins exert a suppressive effect on CD8 cytotoxic lymphocytes, CD4 effectors, and NK cells while stimulating immune suppressor cells like Tregs and MDSCs (37). IDO1 and IDO2 were both downregulated in OL vs. normal tissues (albeit not significantly downregulated; Supplementary Table S1). IDO1 and IDO2 were both upregulated in OM vs. normal tissues (albeit not significantly upregulated; Supplementary Table S2). When comparing OM vs. OL, IDO2 was significantly overexpressed in OM (Table 3).

The immunosuppressive receptor, TIGIT is significantly overexpressed in both OM and OL relative to normal tissues (Tables 1, 2). TIGIT expression can be found on CD4 cells (including Tregs), NK cells, and or CD8 cells. Targeting TIGIT is an option for treating other advanced, cancers and based on these results may be an option for canine melanoma (38, 39). Overall, the data shows that the expression of immune checkpoints was significantly higher in both OL and OM tumors compared to normal tissues, with the OM type demonstrating even higher expression levels of specific immune checkpoints (PD1, IDO2), and lower expression of PDL2 (Table 3).

In addition to the global analysis, one of the OM samples, M337-A2, is different from the rest of the OM group (Figures 2, 3). This oral tumor expresses immune blockade (IDO2) but unlike other OM tumors, conserves interferon response (IRF5 and IRF8), TLR activation (TLR2 and TLR4), and active immune cell markers (CD4). Of note, this was the only tumor in the group documented to have partial bone involvement. Analyzing the heat map for the OL group, four dogs (L794, L600, L080, and L614), had altered expression of the first 57 genes, starting with SPP1 and ending with CCR1, relative to the other OL dogs (Figure 1). This expression pattern suggests a lower inflammatory profile for these four tumors in comparison to the rest of OL tumors (including lower CD4, CD14, CD22, CD37, CD48, CD63, CD68,TLR2, TLR8, cCCL3, chemokines receptor CCR1, and Osteopontin Gene SSP1). All four dogs had tumors located at the hard palate and or lip. Consequently, tumor location might at least partially dictate expression of markers.

## Discussion

Our study has limitations primarily due to its retrospective aspect, the reduced number of samples available, and the absence of strict patient follow-up. In the future, we recommend implementing rigorous enrollment of patients with advanced staging procedures and ensuring regular follow-up. A comprehensive pathology evaluation and genetic investigation should be performed to support these findings further.

The transcriptome analysis of oral melanoma suggests the presence of immune effectors such as NK and CD4 cells, which might be suppressed by negative regulators or immune checkpoints such as the PD1/PDL1/PDL2 axis, TIGIT, or IDO2. Our results suggest that as the disease progresses toward metastasis, some negative regulators increase further, including PD1, PDL1, and IDO2. As a result, exploring immune checkpoint inhibitors as potential treatment may represent a therapeutic opportunity in the veterinary world once the disease is diagnosed and the drugs available, similar to human melanoma. Immune checkpoint-targeted therapy has been a groundbreaking advancement in human cancer therapy over the past decade. The use of antibodies against these immune checkpoints has significantly improved the prognosis of numerous cancers in humans (36). Dog patients would benefit from receiving ICI inhibitors when available in the veterinary world as soon as the disease is diagnosed. Checkpoint inhibitors against immune exhaustion TIGIT could be of particular therapeutic interest for oral melanoma as it was significantly overexpressed in both the OM and OL groups relative to normal tissue in this study.

Our results suggest reduced macrophage infiltration in OM relative to OL tumors, including reduced expression of CD14 in OM relative to both OL and normal tissue. Moreover, there is a low expression of TLR4, and high expression of IRF4 with OM relative to normal tissue. This suggests any residual macrophage population in OM tumors might represent the M2 immunosuppressive type.

With disease progression, changes within the tumor microenvironment, such as hypoxia and an increase in VEGF production, stimulate the TGF beta pathway and trigger an upregulation in inflammatory cytokines and activated pathways (NOS), contributing to immune exhaustion. In this study, VEGFA expression was significantly upregulated in OM tumors relative to OL tumors. As a result, alternative therapeutic strategies for OM tumors could be explored targeting the microenvironment through hypoxia. In both OM and OL tumors there was downregulation of CEACAM1 relative to normal tissue. Furthermore, CEACAM1 was downregulated further in OM compared with OL tumors, suggesting progressive loss of this marker that is associated with a shift from OL to an OM phenotype. This suggests it could be a useful prognostic marker for canine oral melanoma, similar to previous reports describing its prognostic utility in human cancers. Finally, Oncostatin M (OSM) was found to be significantly overexpressed in OM compared with both OL and normal tissue.

The results of this study suggest reactivating immune T lymphocyte cells with interleukins (e.g., IL-2 and IL-15) and TLR agonists for macrophages could benefit the patient. Similarly, radiotherapy for its “vaccine *in situ* effect” or specific targeted RNA vaccines (SOX 10,) could contribute to enforcing the immune response. To establish the credibility of these hypotheses, further evaluation is necessary considering pathology characteristics, such as cell distribution, density, immunochemistry markers, possibly spatial cell communication networks, and single immune cell function assessed through flow cytometry and single-cell RNA seq strategies instead of Nanostring analysis.

In summary, this study utilized historical fixed samples to evaluate the RNA expression of various clinically relevant immuno-oncology genes in canine melanoma. Various genes were found to be significantly altered in patients with metastatic disease relative to patients with local disease, suggesting targeted therapeutic strategies may differ for these patient groups. Overall, the findings have potential value for guiding further studies in canines and developing immunotherapy strategies for melanoma. Future work will aim to develop a landscape score specific to each melanoma patient, enabling the identification of a tailored therapeutic option based on individual immune profiles.

## Data availability statement

The original contributions presented in the study are publicly available. This data can be found in the NCBI repository under accession number: GSE 228574.

## Ethics statement

Written informed consent was obtained from the owners for the participation of their animals in this study ID PRJNA1014399.

## Author contributions

IV: Conceptualization, Data curation, Formal analysis, Funding acquisition, Investigation, Methodology, Project administration, Resources, Supervision, Validation, Writing – original draft, Writing – review & editing. KB: Investigation, Methodology, Project administration, Writing – original draft. CM-V: Data curation, Formal analysis, Visualization, Writing – review & editing. LL: Conceptualization, Data curation, Investigation, Methodology, Writing – original draft. KK: Supervision, Writing – review & editing. MS: Supervision, Writing – review & editing.
