# Supplementary material for: Single institution study of the immune landscape for canine oral melanoma based on transcriptome analysis of the primary tumor
Source: Front Vet Sci. 2024 Jan 8;10:1285909. doi: 10.3389/fvets.2023.1285909 (PMC10800815; doi:10.3389/fvets.2023.1285909)
Supplement: Supplementary file 1 [file Data_Sheet_1.docx]

Supplementary Table 1. **Gene Expression for Local (OL) Vs. Normal Samples**: A selected group of genes is presented based on the cell type commonly expressing them. In addition, a description of the encoded protein by each gene is also included. On the last two columns, the fold change and adjusted P-values are presented for each gene.

| **Local (OL) VS Normal** | | | | |
| --- | --- | --- | --- | --- |
| **Cell type** | **Gene Symbol** | **Protein Description** | **Fold** | **P-Value Adj.** |
| T cell | CD3G | CD3g molecule, gamma (CD3-TCR complex) | 1 | 1 |
|  | CD4 | CD4 molecule | 15.4079 | 0.0000646 |
|  | IL13 | interleukin 13 | 3.02084 | 0.055796 |
|  | IL15 | interleukin 15 | 1.475 | 0.261696 |
|  | IL18 | interleukin 18 (interferon-gamma-inducing factor) | 1.7146 | 0.242462 |
|  | IL2 | interleukin 2 | 1.08154 | 0.866998 |
|  | IL6 | interleukin 6 (interferon, beta 2) | 1.71595 | 0.596006 |
|  | OSM | oncostatin M | 4.23611 | 0.071032 |
|  | THY1 | Thy-1 cell surface antigen | 1.1546 | 0.792864 |
| NK Cells | GZMA | granzyme A (granzyme 1, cytotoxic T-lymphocyte-associated serine esterase 3) | -1.9 | 0.169328 |
|  | KLRG1 | killer cell lectin-like receptor subfamily G, member 1 | 11.231 | 0.000627 |
|  | NCR3 | natural cytotoxicity triggering receptor 3 | 1.01608 | 0.981908 |
| B cells | BLK | B lymphoid tyrosine kinase | 3.35318 | 0.034682 |
|  | CCR5 | chemokine (C-C motif) receptor 5 | 2.56481 | 0.172193 |
|  | TYROBP | TYRO protein tyrosine kinase binding protein | 5.18089 | 0.001619 |
| Macrophages | CD14 | CD14 molecule | 5.8061 | 0.002133 |
|  | CD163 | CD163 molecule | 1.18915 | 0.754188 |
|  | CD68 | CD68 molecule | 4.89344 | 0.009834 |
|  | CD84 | CD84 molecule | 13.3984 | 0.000931 |
|  | CD86 | CD86 molecule | 2.29261 | 0.053836 |
|  | CD99 | CD99 molecule | 1.82053 | 0.032208 |
|  | MIF | macrophage migration inhibitory factor | 1.70396 | 0.161814 |
|  | TLR4 | toll-like receptor 4 | -1.01786 | 0.983992 |
|  | TLR9 | toll-like receptor 9 | 1.14005 | 0.790795 |
| Major Histocompatibility Complex (class1) | MR1 | major histocompatibility complex, class I-related | -1.07784 | 0.866262 |
| Major Histocompatibility Complex (class2) | CIITA | class II, major histocompatibility complex, transactivator | -1.29277 | 0.65125 |
| Cytokines | IFGGC1 | interferon-inducible GTPase 1-like | 1.24429 | 0.827346 |
|  | IFI35 | interferon-induced protein 35 | -1.27941 | 0.637384 |
|  | IFIH1 | interferon induced with helicase C domain 1 | -1.30582 | 0.60106 |
|  | IFIT2 | interferon-induced protein with tetratricopeptide repeats 2 | 1.61136 | 0.279404 |
|  | IFNA7 | interferon, alpha 7 | 4.59887 | 0.000105 |
|  | IFNAR2 | interferon (alpha, beta and omega) receptor 2 | 1.55116 | 0.230771 |
|  | IFNB1 | interferon, beta 1, fibroblast | 1.34722 | 0.677505 |
|  | IFNG | interferon gamma | 1 | 1 |
|  | IFNGR1 | interferon gamma receptor 1 | -1.02119 | 0.981908 |
|  | IRF1 | interferon regulatory factor 1 | -1.0671 | 0.942922 |
|  | IRF2 | interferon regulatory factor 2 | -1.24523 | 0.835814 |
|  | IRF3 | interferon regulatory factor 3 | -1.05075 | 0.978972 |
|  | IRF4 | interferon regulatory factor 4 | 2.37004 | 0.174236 |
|  | IRF5 | interferon regulatory factor 5 | -1.25665 | 0.666342 |
|  | IRF8 | interferon regulatory factor 8 | 1.56818 | 0.344294 |
|  | ISG20 | interferon stimulated exonuclease gene 20kDa | 3.07693 | 0.08175 |
|  | TGFB1 | transforming growth factor, beta 1 | 1.38012 | 0.770565 |
|  | TGFB2 | transforming growth factor, beta 2 | -1.68345 | 0.351465 |
|  | SMAD3 | SMAD family member 3 | -1.85354 | 0.165197 |
| Melanoma Cells | KIT | v-kit Hardy-Zuckerman 4 feline sarcoma viral oncogene homolog | 1.88333 | 0.342444 |
|  | ANXA1 | annexin A1 | -2.06285 | 0.123615 |
|  | BCL2 | B-cell CLL/lymphoma 2 | 2.46065 | 0.002709 |
|  | BRAF | v-raf murine sarcoma viral oncogene homolog B1 | -1.31697 | 0.020664 |
|  | CDKN1A | cyclin-dependent kinase inhibitor 1A (p21, Cip1) | 1.28935 | 0.671421 |
|  | CEACAM1 | carcinoembryonic antigen-related cell adhesion molecule 1 | -3.42594 | 0.006294 |
|  | DMBT1 | deleted in malignant brain tumors 1 | -3.51969 | 0.538708 |
|  | ICAM3 | intercellular adhesion molecule 3 | 2.30855 | 0.144581 |
|  | MCAM | melanoma cell adhesion molecule | -2.39599 | 0.023284 |
|  | MKI67 | antigen identified by monoclonal antibody Ki-67 | -1.37039 | 0.42337 |
|  | NOS2 | nitric oxide synthase 2, inducible | 2.37963 | 0.090197 |
|  | PARP1 | poly (ADP-ribose) polymerase 1 | 1.38996 | 0.78553 |
|  | PCNA | proliferating cell nuclear antigen | 1.54276 | 0.155124 |
|  | PTGS2 | prostaglandin-endoperoxide synthase 2 (prostaglandin G/H synthase and cyclooxygenase) | 1.40756 | 0.639648 |
|  | S100A10 | S100 calcium binding protein A10 | -1.60435 | 0.586854 |
|  | S100A12 | S100 calcium binding protein A12 | 1.02021 | 0.98748 |
|  | S100A4 | S100 calcium binding protein A4 | -1.0045 | 0.994353 |
|  | S100A8 | S100 calcium binding protein A8 | -2.27609 | 0.353614 |
|  | S100A9 | S100 calcium binding protein A9 | -2.95793 | 0.20348 |
|  | SOX10 | SRY (sex determining region Y)-box 10 | 26.6667 | 0.000106 |
|  | TERT | telomerase reverse transcriptase | 1 | 1 |
|  | TP63 | tumor protein p63 | -1.22335 | 0.800843 |
|  | TP53 | tumor protein p53 | -1.2368 | 0.394088 |
|  | VEGFA | vascular endothelial growth factor A | -1.96126 | 0.273357 |
| Immune checkpoints | CTLA4 | cytotoxic T-lymphocyte-associated protein 4 | 1.16071 | 0.78553 |
|  | PDCD1 | programmed cell death 1 | -1.22376 | 0.700311 |
|  | CD274 | Programmed cell death ligand 1 | 2.58523 | 0.0637 |
|  | IDO1 | indoleamine 2,3-dioxygenase 1 | -1.07107 | 0.972316 |
|  | IDO2 | indoleamine 2,3-dioxygenase 2 | -1.52967 | 0.252377 |
|  | LAG3 | lymphocyte-activation gene 3 | -1.05763 | 0.907987 |
|  | TIGIT | T cell immunoreceptor with Ig and ITIM domains | 3.05555 | 0.04822 |
|  | PDCD1LG2 | programmed cell death 1 ligand 2 | 5.49496 | 0.006176 |

Supplementary Table 2. **Gene Expression for Metastatic (OM) Vs Normal Samples**: A selected group of genes is presented based on the cell type commonly expressing them. In addition, a description of the encoded protein by each gene is also included. On the last two columns, the fold change and adjusted P-values are presented for each gene.

| **Metastatic (OM) Vs Normal** | | | | |
| --- | --- | --- | --- | --- |
| **Cell Type** | **Gene Symbol** | **Gene encoding protein** | **Fold** | **P-Value Adj.** |
| T cell | CD3G | CD3g molecule, gamma (CD3-TCR complex) | 1 | 1 |
|  | CD4 | CD4 molecule | 4.38595 | 0.015951 |
|  | IL13 | interleukin 13 | 3.30358 | 0.057239 |
|  | IL15 | interleukin 15 | 1.07333 | 0.853991 |
|  | IL18 | interleukin 18 (interferon-gamma-inducing factor) | -1.38367 | 0.509739 |
|  | IL2 | interleukin 2 | -1.29796 | 0.466943 |
|  | IL6 | interleukin 6 (interferon, beta 2) | 6.1559 | 0.050494 |
|  | OSM | oncostatin M | 40.1851 | 0.000374 |
|  | THY1 | Thy-1 cell surface antigen | -1.90851 | 0.132521 |
| NK Cells | GZMA | granzyme A (granzyme 1, cytotoxic T-lymphocyte-associated serine esterase 3) | 1.33333 | 0.554635 |
|  | KLRG1 | killer cell lectin-like receptor subfamily G, member 1 | 1.17424 | 0.825514 |
|  | NCR3 | natural cytotoxicity triggering receptor 3 | 1.96637 | 0.049649 |
| B cells | BLK | B lymphoid tyrosine kinase | 4.32539 | 0.019839 |
|  | CCR5 | chemokine (C-C motif) receptor 5 | 2.57407 | 0.187818 |
|  | TYROBP | TYRO protein tyrosine kinase binding protein | 1.2257 | 0.690197 |
| Macrophages | CD14 | CD14 molecule | 1.19231 | 0.760002 |
|  | CD163 | CD163 molecule | -2.14773 | 0.078595 |
|  | CD68 | CD68 molecule | 1.12436 | 0.857005 |
|  | CD84 | CD84 molecule | 2.00521 | 0.344814 |
|  | CD86 | CD86 molecule | -2.07442 | 0.131943 |
|  | CD99 | CD99 molecule | -2.3836 | 0.008101 |
|  | MIF | macrophage migration inhibitory factor | 2.59845 | 0.023702 |
|  | TLR4 | toll-like receptor 4 | -2.79411 | 0.021418 |
|  | TLR9 | toll-like receptor 9 | 2.61574 | 0.012437 |
| Major Histocompatibility Complex (class1) | MR1 | major histocompatibility complex, class I-related | 1.62222 | 0.136218 |
| Major Histocompatibility Complex (class2) | CIITA | class II, major histocompatibility complex, transactivator | 1.25386 | 0.639642 |
| Cytokines | IFGGC1 | interferon-inducible GTPase 1-like | 4.17808 | 0.058554 |
|  | IFI35 | interferon-induced protein 35 | 1.52874 | 0.328437 |
|  | IFIH1 | interferon induced with helicase C domain 1 | -1.87751 | 0.167162 |
|  | IFIT2 | interferon-induced protein with tetratricopeptide repeats 2 | 1.17257 | 0.734553 |
|  | IFNA7 | interferon, alpha 7 | 7.10178 | 2.42E-05 |
|  | IFNAR2 | interferon (alpha, beta and omega) receptor 2 | 1.25413 | 0.564039 |
|  | IFNB1 | interferon, beta 1, fibroblast | 1.13889 | 0.83596 |
|  | IFNG | interferon gamma | 1 | 1 |
|  | IFNGR1 | interferon gamma receptor 1 | -2.09767 | 0.116659 |
|  | IRF1 | interferon regulatory factor 1 | 1.65904 | 0.344725 |
|  | IRF2 | interferon regulatory factor 2 | -3.23156 | 0.145898 |
|  | IRF3 | interferon regulatory factor 3 | -1.72226 | 0.561709 |
|  | IRF4 | interferon regulatory factor 4 | 6.83485 | 0.00872 |
|  | IRF5 | interferon regulatory factor 5 | -4.06668 | 0.008101 |
|  | IRF8 | interferon regulatory factor 8 | -1.62963 | 0.308971 |
|  | ISG20 | interferon stimulated exonuclease gene 20kDa | 6.39423 | 0.012437 |
|  | TGFB1 | transforming growth factor, beta 1 | -2.12088 | 0.35564 |
|  | TGFB2 | transforming growth factor, beta 2 | 1.30484 | 0.64187 |
|  | SMAD3 | SMAD family member 3 | -1.03683 | 0.935811 |
| Melanoma | KIT | v-kit Hardy-Zuckerman 4 feline sarcoma viral oncogene homolog | 2.76667 | 0.140584 |
|  | ANXA1 | annexin A1 | -5.8559 | 0.002254 |
|  | BCL2 | B-cell CLL/lymphoma 2 | -1.40668 | 0.24438 |
|  | BRAF | v-raf murine sarcoma viral oncogene homolog B1 | -2.21539 | 2.42E-05 |
|  | CDKN1A | cyclin-dependent kinase inhibitor 1A (p21, Cip1) | -1.01311 | 0.979673 |
|  | CEACAM1 | carcinoembryonic antigen-related cell adhesion molecule 1 | -9.41297 | 0.000127 |
|  | DMBT1 | deleted in malignant brain tumors 1 | -3.85695 | 0.48071 |
|  | ICAM3 | intercellular adhesion molecule 3 | 3.7162 | 0.035191 |
|  | MCAM | melanoma cell adhesion molecule | 1.25628 | 0.573319 |
|  | MKI67 | antigen identified by monoclonal antibody Ki-67 | 1.22575 | 0.604919 |
|  | NOS2 | nitric oxide synthase 2, inducible | 3.22223 | 0.038384 |
|  | PARP1 | poly (ADP-ribose) polymerase 1 | -1.48292 | 0.665848 |
|  | PCNA | proliferating cell nuclear antigen | 1.91176 | 0.049501 |
|  | PTGS2 | prostaglandin-endoperoxide synthase 2 (prostaglandin G/H synthase and cyclooxygenase) | 7.92719 | 0.003146 |
|  | S100A10 | S100 calcium binding protein A10 | -5.74469 | 0.032769 |
|  | S100A12 | S100 calcium binding protein A12 | -5.28425 | 0.073964 |
|  | S100A4 | S100 calcium binding protein A4 | -5.11119 | 0.000385 |
|  | S100A8 | S100 calcium binding protein A8 | -11.5099 | 0.012437 |
|  | S100A9 | S100 calcium binding protein A9 | -14.2991 | 0.007404 |
|  | SOX10 | SRY (sex determining region Y)-box 10 | 48.6906 | 4.25E-05 |
|  | TERT | telomerase reverse transcriptase | 1 | 1 |
|  | TP53 | tumor protein p53 | 1.09917 | 0.705857 |
|  | TP63 | tumor protein p63 | -4.62351 | 0.018215 |
|  | VEGFA | vascular endothelial growth factor A | 2.67311 | 0.133774 |
| Immune checkpoints | CTLA4 | cytotoxic T-lymphocyte-associated protein 4 | 1.73469 | 0.167162 |
|  | PDCD1 | programmed cell death 1 | 3.24433 | 0.010195 |
|  | CD274 | Programmed cell death ligand 1 | 3.57006 | 0.025641 |
|  | IDO1 | indoleamine 2,3-dioxygenase 1 | 1.79012 | 0.489053 |
|  | IDO2 | indoleamine 2,3-dioxygenase 2 | 1.85345 | 0.116659 |
|  | LAG3 | lymphocyte-activation gene 3 | 1.57051 | 0.161092 |
|  | TIGIT | T cell immunoreceptor with Ig and ITIM domains | 5.60184 | 0.006844 |
|  | PDCD1LG2 | programmed cell death 1 ligand 2 | -1.08364 | 0.903988 |

Supplementary Table 3. **Gene Expression for Metastatic (OM) Vs Local (OL) Samples**: A selected group of genes is presented based on the cell type commonly expressing them. In addition, a description of the encoded protein by each gene is also included. On the last two columns, the fold change and adjusted P-values are presented for each gene.

| **Metastatic (OM) Vs Local (OL)** | | | | |
| --- | --- | --- | --- | --- |
| **Cell type** | **Gene Symbol** | **Gene encoding protein** | **Fold** | **P-Value Adj.** |
| T cell | CD3G | CD3g molecule, gamma (CD3-TCR complex) | 1 | 1 |
|  | CD4 | CD4 molecule | -3.51299 | 0.015868 |
|  | IL13 | interleukin 13 | 1.0936 | 0.857903 |
|  | IL15 | interleukin 15 | -1.37422 | 0.307002 |
|  | IL18 | interleukin 18 (interferon-gamma-inducing factor) | -2.37245 | 0.048949 |
|  | IL2 | interleukin 2 | -1.40379 | 0.277271 |
|  | IL6 | interleukin 6 (interferon, beta 2) | 3.58748 | 0.105325 |
|  | OSM | oncostatin M | 9.48633 | 0.003424 |
|  | THY1 | Thy-1 cell surface antigen | -2.20355 | 0.036482 |
| NK Cells | GZMA | granzyme A (granzyme 1, cytotoxic T-lymphocyte-associated serine esterase 3) | 2.53334 | 0.036301 |
|  | KLRG1 | killer cell lectin-like receptor subfamily G, member 1 | -9.5645 | 0.001186 |
|  | NCR3 | natural cytotoxicity triggering receptor 3 | 1.93525 | 0.027144 |
| B cells | BLK | B lymphoid tyrosine kinase | 1.28994 | 0.598774 |
|  | CCR5 | chemokine (C-C motif) receptor 5 | 1.00361 | 0.997911 |
|  | TYROBP | TYRO protein tyrosine kinase binding protein | -4.22687 | 0.003424 |
| Macrophages | CD14 | CD14 molecule | -4.86962 | 0.003424 |
|  | CD163 | CD163 molecule | -2.55398 | 0.017287 |
|  | CD68 | CD68 molecule | -4.35219 | 0.011847 |
|  | CD84 | CD84 molecule | -6.68181 | 0.005718 |
|  | CD86 | CD86 molecule | -4.75581 | 0.001295 |
|  | CD99 | CD99 molecule | -4.33941 | 3.70E-05 |
|  | MIF | macrophage migration inhibitory factor | 1.52495 | 0.221332 |
|  | TLR4 | toll-like receptor 4 | -2.7451 | 0.011192 |
|  | TLR9 | toll-like receptor 9 | 2.29442 | 0.009216 |
| Major Histocompatibility Complex (class1) | MR1 | major histocompatibility complex, class I-related | 1.7485 | 0.047368 |
| Major Histocompatibility Complex (class2) | CIITA | class II, major histocompatibility complex, transactivator | 1.62095 | 0.238524 |
| Cytokines | IFGGC1 | interferon-inducible GTPase 1-like | 3.35781 | 0.061669 |
|  | IFI35 | interferon-induced protein 35 | 1.95588 | 0.086191 |
|  | IFIH1 | interferon induced with helicase C domain 1 | -1.4378 | 0.360434 |
|  | IFIT2 | interferon-induced protein with tetratricopeptide repeats 2 | -1.37421 | 0.421508 |
|  | IFNA7 | interferon, alpha 7 | 1.54425 | 0.132375 |
|  | IFNAR2 | interferon (alpha, beta and omega) receptor 2 | -1.23684 | 0.524643 |
|  | IFNB1 | interferon, beta 1, fibroblast | -1.18293 | 0.753032 |
|  | IFNG | interferon gamma | 1 | 1 |
|  | IFNGR1 | interferon gamma receptor 1 | -2.05415 | 0.077038 |
|  | IRF1 | interferon regulatory factor 1 | 1.77036 | 0.223331 |
|  | IRF2 | interferon regulatory factor 2 | -2.59515 | 0.169026 |
|  | IRF3 | interferon regulatory factor 3 | -1.63907 | 0.54708 |
|  | IRF4 | interferon regulatory factor 4 | 2.88388 | 0.070257 |
|  | IRF5 | interferon regulatory factor 5 | -3.23611 | 0.011934 |
|  | IRF8 | interferon regulatory factor 8 | -2.55556 | 0.035517 |
|  | ISG20 | interferon stimulated exonuclease gene 20kDa | 2.07812 | 0.20182 |
|  | TGFB1 | transforming growth factor, beta 1 | -2.92706 | 0.136159 |
|  | TGFB2 | transforming growth factor, beta 2 | 2.19664 | 0.115184 |
|  | SMAD3 | SMAD family member 3 | 1.78769 | 0.154584 |
| Melanoma | KIT | v-kit Hardy-Zuckerman 4 feline sarcoma viral oncogene homolog | 1.46903 | 0.518765 |
|  | ANXA1 | annexin A1 | -2.83874 | 0.022851 |
|  | BCL2 | B-cell CLL/lymphoma 2 | -3.46134 | 0.00013 |
|  | BRAF | v-raf murine sarcoma viral oncogene homolog B1 | -1.68219 | 0.000982 |
|  | CDKN1A | cyclin-dependent kinase inhibitor 1A (p21, Cip1) | -1.30626 | 0.548226 |
|  | CEACAM1 | carcinoembryonic antigen-related cell adhesion molecule 1 | -2.74756 | 0.016576 |
|  | DMBT1 | deleted in malignant brain tumors 1 | -1.09582 | 0.96588 |
|  | ICAM3 | intercellular adhesion molecule 3 | 1.60976 | 0.331131 |
|  | MCAM | melanoma cell adhesion molecule | 3.01003 | 0.004324 |
|  | MKI67 | antigen identified by monoclonal antibody Ki-67 | 1.67976 | 0.129021 |
|  | NOS2 | nitric oxide synthase 2, inducible | 1.35409 | 0.506341 |
|  | PARP1 | poly (ADP-ribose) polymerase 1 | -2.06121 | 0.345171 |
|  | PCNA | proliferating cell nuclear antigen | 1.23919 | 0.434799 |
|  | PTGS2 | prostaglandin-endoperoxide synthase 2 (prostaglandin G/H synthase and cyclooxygenase) | 5.63185 | 0.003637 |
|  | S100A10 | S100 calcium binding protein A10 | -3.5807 | 0.065662 |
|  | S100A12 | S100 calcium binding protein A12 | -5.39106 | 0.0396 |
|  | S100A4 | S100 calcium binding protein A4 | -5.08829 | 0.000116 |
|  | S100A8 | S100 calcium binding protein A8 | -5.05685 | 0.046783 |
|  | S100A9 | S100 calcium binding protein A9 | -4.83414 | 0.050571 |
|  | SOX10 | SRY (sex determining region Y)-box 10 | 1.82589 | 0.307231 |
|  | TERT | telomerase reverse transcriptase | 1 | 1 |
|  | TP53 | tumor protein p53 | 1.35945 | 0.157129 |
|  | TP63 | tumor protein p63 | -3.77941 | 0.018472 |
|  | VEGFA | vascular endothelial growth factor A | 5.24266 | 0.006493 |
| Immune checkpoints | CTLA4 | cytotoxic T-lymphocyte-associated protein 4 | 1.49451 | 0.235813 |
|  | PDCD1 | programmed cell death 1 | 3.97031 | 0.001295 |
|  | CD274 | Programmed cell death ligand 1 | 1.38095 | 0.490457 |
|  | IDO1 | indoleamine 2,3-dioxygenase 1 | 1.91736 | 0.368744 |
|  | IDO2 | indoleamine 2,3-dioxygenase 2 | 2.83516 | 0.004725 |
|  | LAG3 | lymphocyte-activation gene 3 | 1.66102 | 0.073311 |
|  | TIGIT | T cell immunoreceptor with Ig and ITIM domains | 1.83333 | 0.16992 |
|  | PDCD1LG2 | programmed cell death 1 ligand 2 | -5.95454 | 0.003424 |
