# Supplementary material for: Single institution study of the immune landscape for canine oral melanoma based on transcriptome analysis of the primary tumor
Source: Front Vet Sci. 2024 Jan 8;10:1285909. doi: 10.3389/fvets.2023.1285909 (PMC10800815; doi:10.3389/fvets.2023.1285909)
Supplement: Supplementary file 2 [file Table_1.DOCX]

Table 1: **Clinical Data from Tissue Samples**: Clinical information from the 18 pathology samples, ID samples that start with an L correspond to local tumors, ID samples that start with an M correspond to metastatic samples, and ID samples that start with N correspond to normal tissue samples. Breed, sex, age, and metastasis status are presented with the primary tumor location for all samples. The mitotic index corresponds to the number of cells undergoing mitosis per ten histopathology high power fields as indicated per pathology report from each patient sample.

| **Classification Table of Patient Pathology** | | | | | | | |
| --- | --- | --- | --- | --- | --- | --- | --- |
| ID | Species | Breed | Sex | Age | Tissue Location | Mitotic Index | Metastasis |
| L031-A1 | Canine | Poodle | M | 10 | Left canine gingiva | 0 | No |
| L080-A1 | Canine | Golden Retriever | MN | 7 | Hard palate | 3 | No |
| L088-A1 | Canine | Schnauzer | FS | 11 | Oral Mucosa | 1 | No |
| L194-A1 | Canine | Mixed Breed | FS | 13 | Right maxillary labial gingiva | 0 | No |
| L237-A1 | Canine | Mixed Breed | MN | 10 | Hard palate | 2 | No |
| L562-A1 | Canine | Mixed Breed | F | 6 | Oral Mucosa | 0 | No |
| L600-A1 | Canine | Labrador Retriever | MN | 9 | Right Lip and hard palate | 2 | No |
| L614-A1 | Canine | Mixed Breed | FS | 6 | Hard palate | 1 | No |
| L700-A1 | Canine | Golden Retriever | MN | 11 | Oral Mucosa | 0 | No |
| L794-A1 | Canine | Mixed Breed | MN | 12 | Lip | 3 | No |
| L983-A1 | Canine | Mixed Breed | MN | 11 | Base of tongue | 1 | No |
| L998-A1 | Canine | Mixed Breed | MN | 9 | Oral Mucosa | 0 | No |
| M074-A1 | Canine | Labrador Retriever | MN | 11 | Left Mandibula | 12 | Lungs, Tracheobronchial lymph node, Pulmonary artery |
| M172-A1 | Canine | Coker Spaniel | MN | 12 | Hard palate | 15 | Lymph node, Lung, Liver, Kidney, Heart, Esophagus |
| M286-A1 | Canine | Golden Retriever | FS | 12 | Tonsils | 15 | Regional lymph nodes, lung |
| M337-A2 | Canine | Mixed Breed | FS | 13 | Left Maxilla, bone involvement | 40 | Lungs |
| M570-A1 | Canine | Schnauzer | F | 14 | Jaw region and pharynx | 15 | Lungs |
| M675-A2 | Canine | Scottish Terrier | FS | 12 | Right Maxilla, Peri-orbital | 12 | Lung, Sub Mandibular and medial ileac lymph nodes, mesocolon |
| N1-A1 | Canine | NA | NA | NA | Oral labial mucosa | NA | No |
| N2-A1 | Canine | NA | NA | NA | Oral labial mucosa | NA | No |
| N3-A1 | Canine | NA | NA | NA | Oral labial mucosa | NA | No |
| N4-A1 | Canine | NA | NA | NA | Oral labial mucosa | NA | No |
| N5-A1 | Canine | NA | NA | NA | Oral labial mucosa | NA | No |

Notes: NA=Not available

**Table 2. Significantly Expressed Genes *of* Local (OL) VS Normal**: The genes of local vs. normal that show an adjusted p-value below 0.05 *with a* fold change *considered relevant (+/- 1.5)* are shown in the table. In addition, the *possible associated* cell types expressing the gene, gene symbol, and encoded protein description *are presented.*

| **Significantly Expressed Genes Local (OL) VS Normal** | | | | |
| --- | --- | --- | --- | --- |
| **Cell type** | **Gene Symbol** | **Protein Description** | **Fold** | **P-Value Adj.** |
| T cell | CD4 | CD4 molecule | 15.4079 | 0.0000646 |
| NK cell | KLRG1 | killer cell lectin-like receptor subfamily G, member 1 | 11.231 | 0.000627 |
| B cell | BLK | B lymphoid tyrosine kinase | 3.35318 | 0.034682 |
|  | TYROBP | TYRO protein tyrosine kinase binding protein | 5.18089 | 0.001619 |
| Macrophages | CD14 | CD14 molecule | 5.8061 | 0.002133 |
|  | CD68 | CD68 molecule | 4.89344 | 0.009834 |
|  | CD84 | CD84 molecule | 13.3984 | 0.000931 |
|  | CD99 | CD99 molecule, Protein MIC2 | 1.82053 | 0.032208 |
| Cytokines | IFNA7 | interferon, alpha 7 | 4.59887 | 0.000105 |
| Melanoma cells | BCL2 | B-cell CLL/lymphoma 2 | 2.46065 | 0.002709 |
|  | CEACAM1 | carcinoembryonic antigen-related cell adhesion molecule 1 | -3.42594 | 0.006294 |
|  | MCAM | melanoma cell adhesion molecule | -2.39599 | 0.023284 |
|  | SOX10 | SRY (sex determining region Y)-box 10 | 26.6667 | 0.000106 |
| Immune checkpoints | TIGIT | T cell immunoreceptor with Ig and ITIM domains | 3.05555 | 0.04822 |
|  | PDCD1LG2 | programmed cell death ligand 2 | 5.49496 | 0.006176 |

**Table 3. Significantly Expressed Genes *of* Metastatic (OM) Vs. Normal: The genes of metastatic vs. normal that show an adjusted p-value** below 0.05 *with a* fold change *considered relevant (+/- 1.5)* are shown in the table. In addition, the *possible associated* cell types expressing the gene, gene symbol, and encoded protein description *are presented.*

| **Significantly Expressed Genes Metastatic (OM) Vs Normal** | | | | |
| --- | --- | --- | --- | --- |
| **Cell type** | **Gene Symbol** | **Protein Description** | **Fold** | **P-Value Adj.** |
| T cell | CD4 | CD4 molecule | 4.38595 | 0.015951 |
|  | OSM | oncostatin M | 40.1851 | 0.000374 |
| NK cell | NCR3 | natural cytotoxicity triggering receptor 3 | 1.96637 | 0.049649 |
| B cells | BLK | B lymphoid tyrosine kinase | 4.32539 | 0.019839 |
| Macrophages | CD99 | CD99 molecule, MIC2 | -2.3836 | 0.008101 |
|  | MIF | macrophage migration inhibitory factor | 2.59845 | 0.023702 |
|  | TLR4 | toll-like receptor 4 | -2.79411 | 0.021418 |
|  | TLR9 | toll-like receptor 9 | 2.61574 | 0.012437 |
| Cytokines | IFNA7 | interferon, alpha 7 | 7.10178 | 0.0000242 |
|  | IRF4 | interferon regulatory factor 4 | 6.83485 | 0.00872 |
|  | IRF5 | interferon regulatory factor 5 | -4.06668 | 0.008101 |
|  | ISG20 | interferon stimulated exonuclease gene 20kDa | 6.39423 | 0.012437 |
| Melanoma cells | ANXA1 | annexin A1 | -5.8559 | 0.002254 |
|  | BRAF | v-raf murine sarcoma viral oncogene homolog B1 | -2.21539 | 0.0000242 |
|  | CEACAM1 | carcinoembryonic antigen-related cell adhesion molecule 1 | -9.41297 | 0.000127 |
|  | ICAM3 | intercellular adhesion molecule 3 | 3.7162 | 0.035191 |
|  | NOS2 | nitric oxide synthase 2, inducible | 3.22223 | 0.038384 |
|  | PCNA | proliferating cell nuclear antigen | 1.91176 | 0.049501 |
|  | PTGS2 | prostaglandin-endoperoxide synthase 2 (prostaglandin G/H synthase and cyclooxygenase-2) | 7.92719 | 0.003146 |
|  | S100A10 | S100 calcium binding protein A10 | -5.74469 | 0.032769 |
|  | S100A4 | S100 calcium binding protein A4 | -5.11119 | 0.000385 |
|  | S100A8 | S100 calcium binding protein A8 | -11.5099 | 0.012437 |
|  | S100A9 | S100 calcium binding protein A9 | -14.2991 | 0.007404 |
|  | SOX10 | SRY (sex determining region Y)-box 10 | 48.6906 | 0.0000425 |
|  | TP63 | tumor protein p63 | -4.62351 | 0.018215 |
| Immune checkpoints | PDCD1 | programmed cell death 1 | 3.24433 | 0.010195 |
|  | CD274 | Programmed cell death ligand 1 (PDL1) | 3.57006 | 0.025641 |
|  | TIGIT | T cell immunoreceptor with Ig and ITIM domains | 5.60184 | 0.006844 |

**Table 4. Significantly Expressed Genes *of* Metastatic (OM) Vs. Local (OL): The genes of metastatic vs. local that show an adjusted p-value** below 0.05 *with a* fold change *considered relevant (+/- 1.5)* are shown in the table. In addition, the *possible associated* cell types expressing the gene, gene symbol, and encoded protein description *are presented.*

| **Significantly Expressed Genes Metastatic (OM) Vs Local (OL)** | | | | |
| --- | --- | --- | --- | --- |
| **Cell type** | **Gene Symbol** | **Protein Description** | **Fold** | **P-Value Adj.** |
| T cell | CD4 | CD4 molecule | -3.51299 | 0.015868 |
|  | IL18 | interleukin 18 (interferon-gamma-inducing factor) | -2.37245 | 0.048949 |
|  | OSM | oncostatin M | 9.48633 | 0.003424 |
|  | THY1 | Thy-1 cell surface antigen | -2.20355 | 0.036482 |
| NK cell | GZMA | granzyme A (granzyme 1, cytotoxic T-lymphocyte-associated serine esterase 3) | 2.53334 | 0.036301 |
|  | KLRG1 | killer cell lectin-like receptor subfamily G, member 1 | -9.5645 | 0.001186 |
|  | NCR3 | natural cytotoxicity triggering receptor 3 | 1.93525 | 0.027144 |
| B cells | TYROBP | TYRO protein tyrosine kinase binding protein | -4.22687 | 0.003424 |
| Macrophages | CD14 | CD14 molecule | -4.86962 | 0.003424 |
|  | CD163 | CD163 molecule | -2.55398 | 0.017287 |
|  | CD68 | CD68 molecule | -4.35219 | 0.011847 |
|  | CD84 | CD84 molecule | -6.68181 | 0.005718 |
|  | CD86 | CD86 molecule | -4.75581 | 0.001295 |
|  | TLR4 | toll-like receptor 4 | -2.7451 | 0.011192 |
|  | TLR9 | toll-like receptor 9 | 2.29442 | 0.009216 |
| Major Histocompatibility Complex (class1) | MR1 | major histocompatibility complex, class I-related | 1.7485 | 0.047368 |
| Cytokines | IRF5 | interferon regulatory factor 5 | -3.23611 | 0.011934 |
|  | IRF8 | interferon regulatory factor 8 | -2.55556 | 0.035517 |
| Melanoma cells | ANXA1 | annexin A1 | -2.83874 | 0.022851 |
|  | BCL2 | B-cell CLL/lymphoma 2 | -3.46134 | 0.00013 |
|  | BRAF | v-raf murine sarcoma viral oncogene homolog B1 | -1.68219 | 0.000982 |
|  | CEACAM1 | carcinoembryonic antigen-related cell adhesion molecule 1 | -2.74756 | 0.016576 |
|  | MCAM | melanoma cell adhesion molecule | 3.01003 | 0.004324 |
|  | PTGS2 | prostaglandin-endoperoxide synthase 2 (prostaglandin G/H synthase and cyclooxygenase-2) | 5.63185 | 0.003637 |
|  | S100A12 | S100 calcium binding protein A12 | -5.39106 | 0.0396 |
|  | S100A8 | S100 calcium binding protein A8 | -5.05685 | 0.046783 |
|  | S100A9 | S100 calcium binding protein A9 | -4.83414 | 0.050571 |
|  | TP63 | tumor protein p63 | -3.77941 | 0.018472 |
|  | VEGFA | vascular endothelial growth factor A | 5.24266 | 0.006493 |
| Immune checkpoints | PDCD1 (PD1) | programmed cell death 1 | 3.97031 | 0.001295 |
|  | IDO2 | indoleamine 2,3-dioxygenase 2 | 2.83516 | 0.004725 |
|  | PDCD1LG2 | programmed cell death ligand 2 | -5.95454 | 0.003424 |
